# Anne O'Tate: A tool to support user-driven summarization, drill-down and browsing of PubMed search results

**DOI:** 10.1186/1747-5333-3-2

**Published:** 2008-02-15

**Authors:** Neil R Smalheiser, Wei Zhou, Vetle I Torvik

**Affiliations:** 1 Department of Psychiatry and Psychiatric Institute, MC912, University of Illinois at Chicago, Chicago, IL 60612, USA; 2 Department of Computer Science, University of Illinois at Chicago, Chicago, IL 60607, USA

## Abstract

**Background:**

PubMed is designed to provide rapid, comprehensive retrieval of papers that discuss a given topic. However, because PubMed does not organize the search output further, it is difficult for users to grasp an overview of the retrieved literature according to non-topical dimensions, to drill-down to find individual articles relevant to a particular individual's need, or to browse the collection.

**Results:**

In this paper, we present Anne O'Tate, a web-based tool that processes articles retrieved from PubMed and displays multiple aspects of the articles to the user, according to pre-defined categories such as the "most important" words found in titles or abstracts; topics; journals; authors; publication years; and affiliations. Clicking on a given item opens a new window that displays all papers that contain that item. One can navigate by drilling down through the categories progressively, e.g., one can first restrict the articles according to author name and then restrict that subset by affiliation. Alternatively, one can expand small sets of articles to display the most closely related articles. We also implemented a novel cluster-by-topic method that generates a concise set of topics covering most of the retrieved articles.

**Conclusion:**

Anne O'Tate is an integrated, generic tool for summarization, drill-down and browsing of PubMed search results that accommodates a wide range of biomedical users and needs. It can be accessed at [4]. Peer review and editorial matters for this article were handled by Aaron Cohen.

## 1. Background

Anne O'Tate was developed as a part of the Arrowsmith project [1-4], which has been developing informatics tools for advanced text mining of the biomedical literature. We sought to create a tool for carrying out PubMed searches [5] that did not require the user to progressively reformulate the initial query; that would assist the user in finding the most relevant articles quickly and efficiently; and that would summarize the salient features of a given set of articles – e.g., given a set of articles discussing gene X, to give a list of diseases that gene X has been studied in, or given a set of articles on disease Y, to give a list of symptoms that have been described in that disease. The present paper describes the current implementation of Anne O'Tate, which is used routinely by our group for conducting PubMed searches. The tool has been placed on the Arrowsmith homepage [4] as a free, public web-based service.

## 2. Implementation

### 2.1 Query interface

The PubMed query interface [5] was imported into the Anne O'Tate web page, so that when a user types in a query, it is sent to PubMed using the NCBI E-Utilities (ESearch and EFetch) [6] to obtain the PubMed IDs, and thereby takes advantage of the pre-processing that occurs within PubMed. Given the set of PubMed IDs, articles are looked up in a local MEDLINE/PubMed database; for articles not included in the local database, E-Utilities are used to download the records of those (generally very recent) articles. There is no restriction on the number of articles retrieved from PubMed and displayed initially to the user. However, to limit the computational load on the system, a limit was placed on the number of papers that are processed further (as discussed below). At present, the default limit is set to process further only the 25,000 most recent articles of a given query.

### 2.2 MEDLINE term database

A database of terms was created including all of the words and phrases [*n*-grams (*n *= 1,2,3)] that occur in the title of at least one article in MEDLINE. A simple tokenizer (to remove sentence delimiters and change the text to lower case) and a stemmer (to handle plurals) have been applied [7]. In total, 15.5 million terms were extracted. Document frequency is defined as the number of different articles in MEDLINE that contain the term in either title or abstract. Each term in an article is counted only once, even though it may occur several times in that article. We intend to update the term database yearly.

#### Semantic categories

Terms were run through the NIH MetaMap program (MMTx version 2.0) [8] to assign each term to one or more semantic categories, if possible, as defined by the Unified Medical Language System (UMLS). The 134 semantic categories were grouped into ~15 super-categories as outlined in [9]. (For example, a number of individual semantic categories such as Hazardous or Poisonous Substance, Hormone, and Immunologic Factor were subsumed under the super-category of Chemicals & Drugs.) Because MetaMap cannot optimally recognize terms out of context, and because at the time certain terms were poorly represented in the UMLS, including neuroanatomical terms and gene/protein names, the NeuroNames vocabulary [10] and a list of predicted gene and protein names extracted from Entrez Gene [11] were added as complementary semantic categories. Anne O'Tate allows users to restrict important words (see below) or MeSH terms to any of the 15 super-categories or to any of the individual semantic categories therein; alternatively, they can retain all terms that mapped to at least one semantic category while discarding terms that failed to map at all.

### 2.3 Anne O'Tate categories

#### 1. Important words

Important words distinguish a specific literature *L *from the rest of MEDLINE. Important words of a literature should occur significantly more frequently within the literature than overall in MEDLINE. That is, they should show high **enrichment**, forming a literature-specific vocabulary that is similar to the concept of a domain sub-language [12]. At the same time, important words should ideally occur in a high proportion of the articles in literature *L *(i.e., should have high **coverage**).

To create a list of words that are highly enriched within a given retrieved literature *L *relative to MEDLINE as a whole, the null hypothesis is that *L *and a given word *t *are independent of each other, in which case the number of articles within that literature that contain the word will follow the hyper-geometric distribution. Words occurring one time in *L *were discarded from consideration. Given *n*, the number of articles in MEDLINE containing word *t *in title or abstract; and *N*, the number of articles in MEDLINE, we calculated the parameter Ent. This parameter is related to the probability that word *t *occurs at or above the observed document frequency (*f*) in *L*. Specifically, the Ent score is equal to the t-statistic; for example, Ent = 3 is equivalent to the statement that *t *is significantly enriched in *L *at p = 0.001). When *N *is large compared to *|L|*, Ent is approximately:

$$Ent=-ln\left[\sum_{x=f}^{\left|L\right|}\frac{e^{\lambda }\lambda^{x}}{x!}\right]$$

where *λ *= |*L*| * *n*/N is the expected value of *f*.

For each retrieved literature *L*, we created a list of all words that had a very high enrichment value (i.e. p ≤ 0.001) as calculated above. These were then displayed in order of their relative "importance score" which takes into account both enrichment and coverage, using the formula: Importance = (f/|L|)^2^/n.

#### 2. Topics (i.e., Medical Subject Headings)

Articles in MEDLINE are indexed by Medical Subject Headings (MeSH); these are annotated by expert biologists, follow a standardized hierarchical set of terminology, and are used to describe the main topics discussed [13]. We display the MeSH terms used in the PubMed search output (stoplisting the 20 most frequent MeSH terms in MEDLINE from consideration as being too general to be useful, such as *Humans*, *Male*, *Female*, etc.).

#### 3. Affiliations

Within the affiliation field, text delimited by commas is extracted, assuming that these correspond to meaningful components such as institutions, departments, cities, states, zip codes, or countries. They were not tokenized or stemmed. In addition, different text segments that always co-occur were displayed together. For example, "Yale University School of Medicine" always co-occurred with "Connecticut". As such, Anne O'Tate put them together as a single affiliation term.

#### 4. Other MEDLINE fields

Anne O'Tate also displays the search results according to other MEDLINE fields, including author names, journals, and year of publication, listed in order of frequency within the PubMed search output. These fields allow users to have a quick overview of the retrieved literature from different perspectives.

### 2.4 Literature expansion

The literature expansion tool was added in order to assist the user when he or she finds themselves examining a very small set of articles after running a PubMed query. This situation may arise for at least 3 reasons: a) The PubMed query may relate to a new or highly specific research area in which few articles are available. b) The query may have been poorly formulated so that most relevant papers were missed. c) The user may have already used the Anne O'Tate tool to drill down a few levels within the initial search output.

The PubMed "related articles" function [14] was employed in batch mode to expand a retrieved literature *L *containing fewer than 50 articles. For each article in *L*, a list of its most related articles is retrieved from PubMed using its Elink utility, and the top 100 are kept. These related articles are pooled, and for each of the related articles in the pool, we ask whether it is related to at least 40% of the articles in *L*. (When *L *contains only 2–4 articles, a related article must be related to at least 2 of them.) There may be hundreds of related articles satisfying these criteria, but we only display the 50-*L *most related articles so that the total number of displayed articles (*L *+ related articles) is equal to 50. The expansion not only provides more relevant articles to the user, but also gives a reasonably big literature for Anne O'Tate to summarize.

### 2.5 The cluster-by-topic function

**Figure 1:**
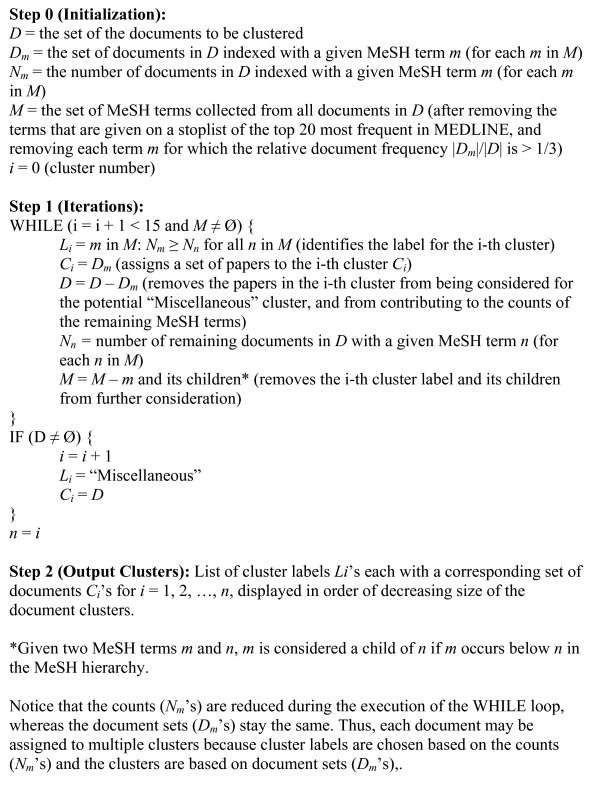
The Cluster-by-topic algorithm.

The goal of cluster-by-topic is to partition the search results coarsely into several clusters according to major topics, giving the user a quick overview of the retrieved literature. Very recent articles not yet indexed by MeSH terms are placed into a cluster called "Most recent articles," whereas older articles not indexed by MeSH (e.g., articles from the 1950s) are placed into another cluster called "Not indexed by topic." For those articles indexed by MeSH terms, a simple and efficient clustering algorithm is applied as described in Fig. 1. With this algorithm, any retrieved literature is split into a small set of no more than 18 clusters (i.e., "Most recent articles", "Not indexed by topic", "Miscellaneous", and up to 15 MeSH-based clusters which are displayed in order of size). Note that an article may fall into several clusters ("soft clustering") since an article usually has several major topics.

## 3. Results

### 3.1 Top ranked important words include important biological concepts

It is interesting to notice that the top ranked important words include many abbreviations and gene/protein symbols. For example, in Table 1, "ad" is the abbreviation for "alzheimer disease"; "abeta" for "amyloid beta-peptide"; "apoe" for "apolipoprotein E"; "app" for "amyloid precursor protein"; "abeta42" for "beta-amyloid 42"; "mmse" for "Mini-Mental State Examination"; and "ps1" for "presenilin-1". Among these abbreviations, "app", "apoe", and "ps1" are gene symbols or aliases.

**Table 1:** Top 20 most important words for the PubMed query "Alzheimer Disease [MeSH Term]".

Rank	Important words	Rank	Important words
1	alzheimer	11	app
2	ad	12	donepezil
3	abeta	13	secretase
4	dementia	14	cognitive
5	amyloid	15	abeta42
6	neurofibrillary	16	mmse
7	tangle	17	neurodegenerative
8	presenilin	18	presenilin-1
9	epsilon4	19	disease
10	apoe	20	ps1

One possible use for the "important words" function is to annotate a collection of genes and proteins according to the major concepts and items discussed regarding each. Each gene or protein can be used as input to a PubMed search, and the retrieved literature is processed to provide a list of the most important words.

### 3.2 Categories defined by MEDLINE fields

**Figure 2:**
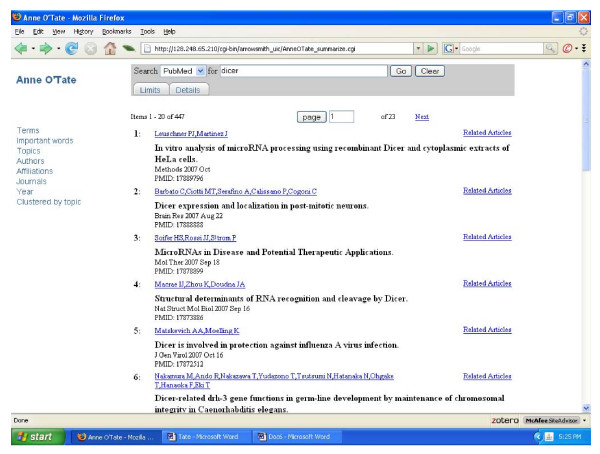
Screenshot of the Anne O'Tate tool returning the PubMed query "dicer."

**Figure 3:**
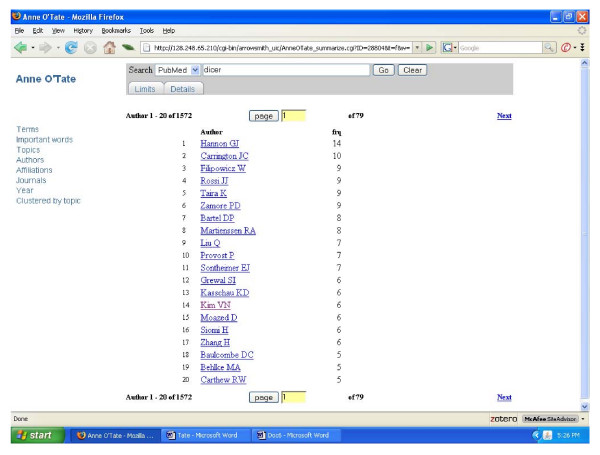
Screenshot of the Anne O'Tate tool displaying a list of the author names mentioned in the set of articles retrieved by the "dicer" query.

**Figure 4:**
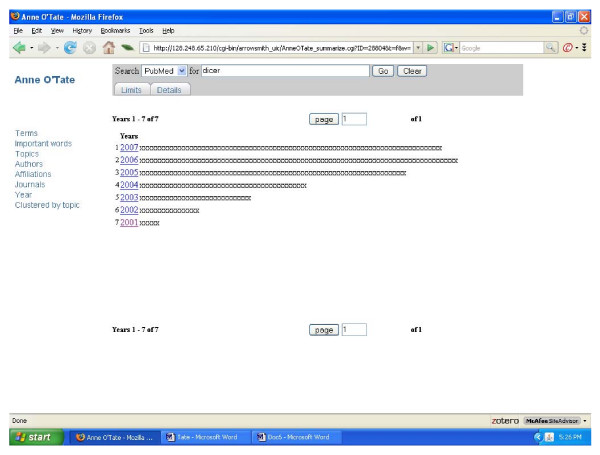
Screenshot of the Anne O'Tate tool displaying a histogram of the publication dates of the set of articles retrieved by the "dicer" query.

To give a typical example of how the MEDLINE fields may assist in categorizing search results for further analysis, consider how a user might seek to gain an overview of the articles that have studied or discussed the RNAse III enzyme dicer, which processes double-stranded RNA to form small inhibitory RNAs and microRNAs. The term "dicer" was inputted and a set of 447 articles were retrieved from PubMed on September 26, 2007 (Fig. 2). Clicking on any of the categories shown at the left provides a thumbnail sketch of that literature: The most frequent author names, co-authors, journals, affiliations, MeSH topics, and the "important words" discussed above. For example, clicking on author name displays a list of the authors who have published the most papers on dicer (Fig. 3). Clicking on Topics or Important Words gives different, complementary views of the most prevalent and important items discussed in this field. Users also have the option of restricting the semantic category(ies) of the displayed terms, topics or important words. Clicking on the Year shows a histogram of the distribution of articles according to publication date (Fig. 4). Clicking on any displayed item, in any category, automatically produces a new, restricted PubMed query and search output in a new window. For example, in fig. 4 one can appreciate that articles on dicer first appeared in 2001; clicking on "2001" allows one to view just the 7 papers that appeared in that year. This effectively allows one to refine and reformulate queries progressively without starting over. At any point in which the resulting search output contains less than 50 articles, an "Expand" button automatically appears which allows one to include the most related articles. In the case of the 2001 dicer articles, the expansion identifies an additional 43 papers (from various years), which represent the articles most similar to the 2001 articles considered as a group. These can be analyzed and processed further at will.

### 3.3 Browsing: search results are clustered into topics with a high coverage

For a person unfamiliar with the dicer literature, browsing the various categories may be a useful way to gain an overview and decide which, if any, articles to examine. However, in order to provide an even more succinct overview, we added a "clustered by topic" button which divides any literature into no more than 18 clusters, i.e., the size of a list that can fit comfortably onto one page.

**Figure 5:**
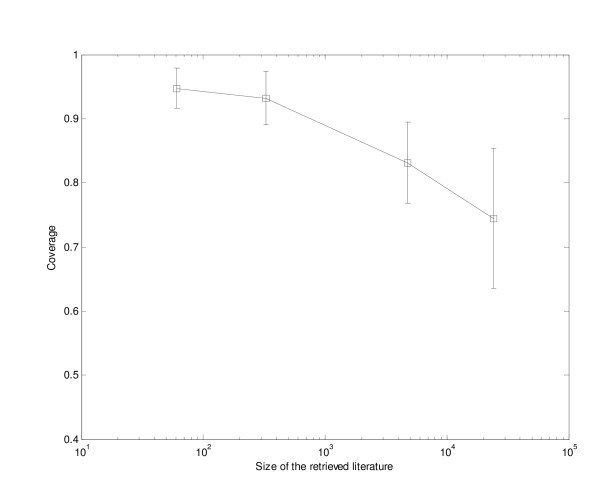
**Coverage of the cluster-by-topic list across a range of queries**. Anonymous queries in the Anne O'Tate query web log were analyzed. For each query, the coverage was computed (i.e., the proportion of MeSH-indexed articles in the PubMed search output that were included in the 15 MeSH-based topical clusters). The results were averaged for retrieved literatures of different size ranges as follows: 0–100 articles, 6 queries; 101–1000 articles, 9 queries; 1001–10000 articles, 9 queries; and >10000 articles, 3 queries.

To evaluate our clustering method, 27 anonymous queries in the Anne O'Tate query web log were analyzed. For each query, the coverage was computed (i.e., the proportion of MeSH-indexed articles in the PubMed search output that were included in the 15 MeSH-based topical clusters). The number of articles for these 27 queries ranged from 40 to 40,000. As shown in Fig. 5, coverage for relatively small literatures was usually > 90%, and was > 70% even for a PubMed query with more than 40,000 articles.

Table 2 gives an example of clustering for a large PubMed query: "Alzheimer Disease [MeSH Term]". One can see that both the cluster-by-topic function (Table 2) and the "important words" function (Table 1) capture many of the major aspects of Alzheimer disease, but there are significant differences as well. For example, the "important words" include specific names such as MMSE (Mini Mental State Examination) and Donepazil (a generic drug name), whereas the cluster-by-topic list includes more general categories, e.g., neuropsychological tests, caregivers, and Parkinson disease (a different but related neurodegenerative disease). Thus, they present the user with differing, and to some extent, complementary perspectives.

**Table 2:** Clustering the search results of the PubMed query "Alzheimer Disease [MeSH Term]" using the cluster-by-topic function.

Rank	Topic	Count*
	Most recent articles	399
1	Aged, 80 and over	6282
2	Brain	4711
3	Amyloid beta-Protein	3983
4	Neuropsychological Tests	2846
5	Cognition Disorders	2454
6	Neurons	2022
7	Apolipoproteins E	1969
8	Dementia	1897
9	Risk Factors	1791
10	Aging	1616
11	Cholinesterase Inhibitors	1535
12	Tau Proteins	1382
13	Membrane Proteins	1289
14	Caregivers	971
15	Parkinson Disease	899

	Not indexed by topic	52

	Miscellaneous	4949

42,671 articles were retrieved from PubMed, 87% of which are included in the 15 MeSH-based topical clusters.

## 4. Discussion

A variety of web-based text mining tools are available that allow users to post-process a PubMed search output according to pre-defined categories (Table 3) [15-29], particularly displaying articles grouped by author names, affiliations, topics, text words and years of publication. Anne O'Tate is perhaps most similar to PubReMiner [24] in that both tools allow the user to input a literature search through the familiar PubMed interface and then proceed immediately to analysis, in contrast to tools such as Meva [22] which require the user to download a file from PubMed and then separately upload it to the Meva server. Both tools employ MeSH terms to define topical categories, in contrast to tools such as ClusterMed [26] whose default clustering strategy is based on title and abstract terms.

**Table 3:** Some currently available web-based tools that allow users to carry out post-processing of PubMed queries.

**Type**	**Site**	**URL**	**Features**
**TYPE 1**: extract relationships and allow for 1) graphical visualization or navigation; or 2) refining queries	AliBaba [15]		Extract relationships between biological objects and map them into a graphical network
	BioIE [16]		Extract informative sentences from retrieved results
	Chilibot [17]		Extract biological relationships from search results
	ConceptLink [18]		Extract relationships between medical concepts and allow graphical visualization
	PubNet [29]		Extract several relationships from the search results and then map them into networks
	XplorMed [20]		Extract dependency relations among words and allow users to refine queries using these words
			
**TYPE 2**: organize results by ontologies or hierarchies	GoPubMed [21]		Sort PubMed query results through Gene Ontology and MeSH hierarchy
	MEVA [22]		Summarize search results according to MeSH hierarchy
	FABLE [23]		Tag gene and protein occurrences in text
	PubReMiner [24]		Categories include words, MeSH, authors, journals, year, substances and country
	PubMed Assistant [25]		Lists MeSH and chemicals, with link-outs to PubMed, Google and Google Scholar
			
**TYPE 3**: cluster articles into categories	Vivísimo ClusterMed [26]		Cluster articles into several categories
	HubMed [27]		Cluster related articles and allow for graphical visualization
			
**TYPE 4**: rank articles	PubFocus [28]		Rank articles by the journal impact factor and volume of forward references
	ReleMed [29]		Rank articles by relevance

Each of the available web-based tools has unique features, and may be preferred for particular users, types of queries or types of analyses. However, Anne O'Tate offers at least 4 unique features that, to our knowledge, are not found in any other tool at present, and that taken together make it a flexible and practical option for summarization, drill-down and browsing of biomedical articles:

First, the current implementation of Anne O'Tate permits analysis of the 25,000 most recent articles retrieved by any PubMed search, which is much larger than can be handled by other tools; this feature makes it an everyday work-horse rather than a prototype. The emphasis on large literatures did not permit us to include computation-intensive visualization capabilities such as are provided by Alibaba [15] or HubMed [27]. However, we were able to include a "clustered by topic" feature that represents the major topics covered by a set of articles in an extremely concise form, by developing a novel clustering algorithm that is computed efficiently and is scalable to very large literatures. This allowed us to cluster tens of thousands of articles in real time, whereas other public interfaces [26] permit users to cluster no more than 500 articles.

Second, search results can be progressively narrowed down by simple clicking, according to any category, allowing one to find articles of interest without needing to modify and re-input the initial query. Thus, Anne O'Tate allows users to direct their attention according to which articles are of greatest interest but does not attempt to predict in advance which articles are likely to be most relevant; this philosophy differs from tools such as HubMed [27] or Relemed [29], which display articles in order of predicted relevance to the input query

Third, the "important words" of the retrieved literature are displayed, with an option to restrict these to user-defined semantic categories. A list of "important words" will avoid displaying many general items commonly discussed throughout MEDLINE (such as gene, protein, human, cell, etc.), and thus is more informative than displaying a simple list of the most frequent words.

Fourth, when the number of displayed articles is less than 50, the user has the option to view additional articles that are most closely related to the existing set considered as a whole. This extends the power of the existing PubMed "related records" feature that finds the most closely related articles relative to a single index article.

In our own experience, the web interface has been a useful, daily tool to enhance routine PubMed searching. Anne O'Tate is freely available as a web-based service with no need for log-ins, passwords or downloads; we invite users to employ Anne O'Tate in their own searches and to provide feedback and suggestions for improving its features and aligning it with the needs of the biomedical community.

## 5. System performance, availability and requirements

Anne O'Tate is currently running on a server with two Xeon 2.4 G processors and 6 GB RAM. Computation time increases linearly to the number of articles to be post-processed. At present, times range from <1 second (to compute the important words for 100 articles) to ~100 seconds (to compute the important words for 25,000 articles containing abstracts).

## Authors' contributions

NS directed the development of Anne O'Tate and wrote part of the paper. WZ programmed Anne O'Tate and its web interface, carried out the experiments described, and wrote part of the paper. VT devised the cluster-by-topic algorithm, created the terminologic databases and maintained the web servers.
